# Prescription Department

**Published:** 1894-05

**Authors:** 


INANITION IN CHILDREN.

℞ Acidi phosphorici dil. ♏xxxij.

Tr. nucis vomicæ, ♏viij.

Vini xerici.

Syr. lactopetinæ vel. simplicis, āā ʒ iv.

Aquæ, q. s. ℥ ij.

M. Sig. Take one teaspoonful three times a day, before meals.

TOOTH AND MOUTH WASH.

℞ Sennine, ʒ ij.

Alcohol, f ℥ iv.

Ol. menth. pip., drops, vj.

Tinct. eucalvpti, f ℥ ss.

Glycerin, f ℥ jss.

M. Sig. Tablespoonful in a wine glassful of water as tooth or mouth wash.

ACUTE CORYZA.

℞ Camphor, gr. xx.
Cocaine mur., gr. xv.
Bismuthi subnit., ℥ ss.
M. Sig. Use as snuff.—*Prof. Waxham in Gross Med. Coll. Bull.*

FOR SHOCK FOLLOWING ABDOMINAL OPERATONS.

By Dr. Edward P. Davis, Demonstrator of Obsterics:

℞ Elixir ammonii valerianat. f ʒ j.

Spirit frumenti f ℥ j.

Aquæ bullient., f ℥ ij.

M. Sig. Give by rectal injection every two hours.

CHRONIC GASTRIC ULCER.

℞. Creasote, ♏ iv.

Aquæ. ℥ vj.

M. Sig. Tablespoonful four times a day.—*Niemeyer.*

HYDROBROMIC COUGH-MIXTURE.

℞ Dil. hydrobromic acid,

Spir. chloroformi. āā ♏ iv.

Syr. squill., ♏ viij.

Camph. tinct. opium, q. s. ʒ ij.

Mix. Dose, ʒ ij.—*Roosvelt Hospital Formulary; The Prescription.*

FOR ECZEMA, HERPES—OTHER SKIN AFFECTIONS.

℞ Pineoline, 2 ozs.
Sig. Apply twice daily.

FOR OBESITY AND FATTY DEGENERATION OF THE HEART.

℞ Phytoline. 2 ozs.
Sig. Ten drops an hour before and after meals in a glass of water (hot if possible).

This prescription can be filled by any retail druggist in the United States.

CHRONIC BRONCHITIS.

℞ Terpin., gr. iv.
Tinct. nucis vom., gtt. ij.
Tinct. opium, gtt. iij.
Glycerin,
Gum, āā enough to make a soft pill.

M. Sig. 1 such pill to be taken four or five times a day.—*Le Prog. Méd.*

ALCOHOLISM.

Dr. T. W. Bath, of Ohio, Ill., puts his patients upon hypodermic injections of strychnine and atropine, beginning with $\frac{1}{20}$ grain strychnine nitrate and $\frac{1}{100}$ grain atropine sulphate. After three days he increases the dose, and at the end of a week again. During the second week two or three daily injections are given of strychnine nitrate, gr. $\frac{1}{10}$, and atropiæ, gr. $\frac{1}{100}$. In the third week the strychnine is reduced to $\frac{3}{40}$ grain, generally maintaining the dose of atropine without change. During the early part of the treatment he orders, as an adjuvant:

℞ Fluid ext. nux vomica, ♏iij.

Tr. capsicum, ♏iii–v.

Fluid ext. digitalis, ♏ss.

Fowler's sol., ♏ss.

Strychnia nitrate, gr. $\frac{1}{100}$–$\frac{1}{75}$.

M. Sig. Five to seven teaspoonfuls per day.

